# Congenital hepatic fibrosis and coexistent retinal macular degeneration: A case report

**DOI:** 10.1097/MD.0000000000016909

**Published:** 2019-08-30

**Authors:** Dezhao Li, Junjie Qin, Shijuan Sun, Xu Li

**Affiliations:** Department of Hepatology, the First Hospital of Jilin University, Changchun, China, Jilin Province, China.

**Keywords:** congenital hepatic fibrosis, ductal plate malformation, portal hypertension, retinal macular degeneration

## Abstract

**Rationale:**

Congenital hepatic fibrosis (CHF) is an autosomal recessive disease characterized by periportal fibrosis, portal hypertension, and renal cystic disease. Essentially, CHF is a variant of fibrocystic disorder in which liver and kidney are commonly affected. Other frequently associated conditions include Caroli syndrome and polycystic kidney disease. CHF is also a known accompaniment in an array of inherited disorders with multiorgan involvement.

**Patient concerns:**

The 20-year-old male patient with declining vision (14 years duration), intermittent gingival bleeding (7 years duration), and abdominal distension (5 years duration), presented with exacerbation of these symptoms during the prior 2 months. The patient had been previously diagnosed with retinal macular degeneration, idiopathic thrombocytopenic purpura, and hepatosplenomegaly.

**Diagnoses:**

Liver biopsy showed disordered hepatic acini and fibrous parenchymal banding, indicative of CHF.

**Interventions:**

After the treatment of diuresis and liver protectants, the clinical symptoms of the patients were improved. We subsequently recommend chromosomal analysis, although the family refused.

**Outcomes:**

Three months after discharge, the patient was followed up by telephone. The patient had obvious abdominal distension and we advised that he should be admitted again. But the family refused.

**Lessons:**

CHF is an AR disease resulting in portal hypertension and often associated with renal malformations. CHF is also linked to a number of other disorders, many of which are ciliopathies. Because the clinical manifestations of CHF are nonspecific or lacking, its diagnosis is problematic, relying largely on liver biopsy. Once CHF is identified, physicians are obligated to investigate other organ systems, particularly a search for neuromuscular, retina or renal involvement. This case underscores the value of radiologic imaging, pathologic examination, and genetic testing in successfully diagnosing a rare disease.

## 1 Introduction

Congenital hepatic fibrosis (CHF) is attributed to a ductal plate malformation in which persistence of immature embryonic bile ducts incites excess proliferation of fibrous tissue in portal areas,^[1,2]^ leading to portal hypertension, splenomegaly, hypersplenism, upper gastrointestinal varices, and ascites. CHF is also frequently associated with ciliopathies (disorders of primary cilia) and the phenotype may involve kidneys, collectively termed hepatorenal fibrocystic disease. Autosomal recessive polycystic kidney disease (AR-PKD) is the most likely concomitant ailment, as opposed to juvenile nephronophthisis, various syndromic conditions (Meckel-Gruber, Bardet-Biedl, Jeune, or Joubert), and related disorders that present with less frequency.^[3,4]^

Herein, we report a 20-year-old man with portal hypertension and retinal macular degeneration whose liver biopsy showed ductal-plate malformation characteristic of CHF. Hepatosplenomegaly and portal hypertension are the chief manifestations of CHF, arising principally in children and young adults.

## 2 Case report

A 20-year-old man was admitted to our facility with declining vision (14 years), intermittent gingival bleeding (7 years), and abdominal distension (5 years), all of which were exacerbated during the prior 2 months. In 2004, his impaired visual acuity was diagnosed as retinal macular degeneration. In 2011, he presented with gingival bleeding and received a diagnosis of idiopathic thrombocytopenic purpura (ITP), hepatosplenomegaly at a local hospital.

The patient was seen at our institution in 2013 for abdominal distension. At that time, his platelet count was low (66 × 10^9^/L), but γ-glutamyltransferase (150.8 U/L) and serum copper levels were normal. Autoimmune antibody and immunoglobulin screens were also negative. However, abdominal computed tomography (CT) showed hepatomegaly, splenomegaly, portal hypertension, and ascites. Results of ophthalmic testing were as follows: right vision, 0.02; left vision, 0.01; right intraocular tension, 19 mmHg (1 mmHg = 0.133 kPa); left intraocular tension, 19 mmHg. Both optic discs were clear, light in color, with somewhat delicate blood vessels. The macular regions reflected gold, foil-like light, compatible with retinal macular degeneration. Bone marrow biopsy confirmed low numbers of megakaryocytes and platelets.

We subsequently recommend chromosomal analysis, although the family refused. Cryptogenic thrombocytopenia and retinal macular degeneration were diagnosed clinically. Two months later, the patient was readmitted to our facility for further evaluation and treatment of worsening abdominal distension, the cause of which was unclear. Obesity, impaired vision, abdominal protuberance, hepatomegaly (1 cm below right costal margin), and splenomegaly were apparent on physical examination. The laboratory test results, including normal liver enzymes, are provided in Table 1.

**Table 1:**
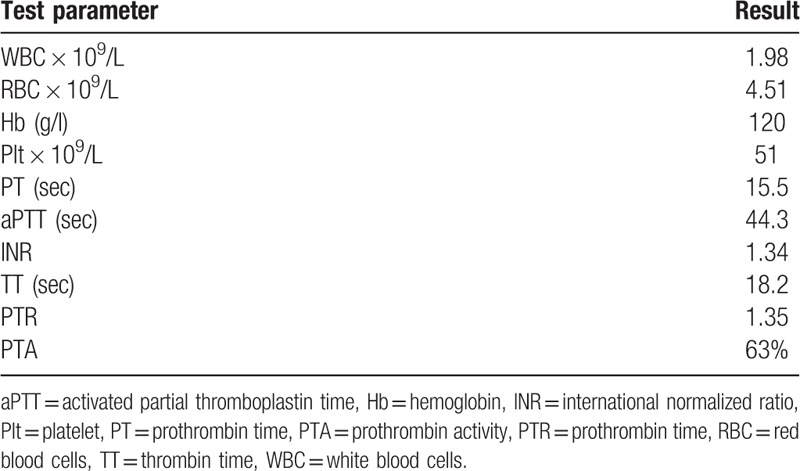
Laboratory test results upon patient readmission.

**Figure 1:**
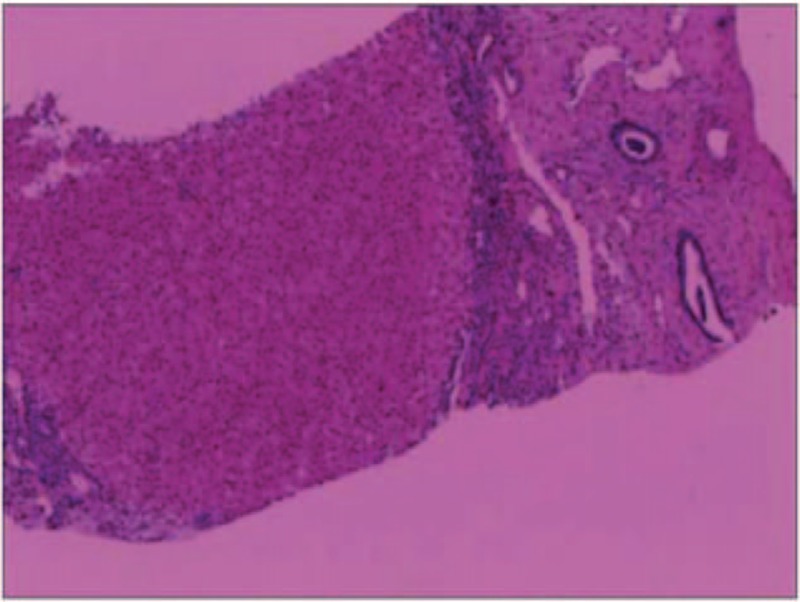
Liver biopsy showing histologic hallmarks of congenital hepatic fibrosis, including a widened portal tract with abnormally formed bile ducts and periportal fibrosis (hematoxylin and eosin stain).

Upper gastrointestinal endoscopy revealed severe esophageal varices, prohibiting further examination. By Doppler ultrasound, the diameter of the main portal vein was 16.5 mm, but its blood flow was unobstructed (interpreted as portal vein widening). Magnetic resonance imaging (MRI) of the abdomen disclosed cirrhosis, a giant spleen (with nodules), and ascites, but no renal lesions were evident. Liver biopsy was performed, demonstrating disordered hepatic acini and fibrous parenchymal banding (Fig. 1). Bile ductular proliferation and dilation were noted, indicative of CHF. Given this unusual clinical presentation and the related histologic findings, a referral for genetic workup was initiated, which the family declined. Three months after discharge, the patient was followed up by telephone. The patient had obvious abdominal distension, and was advised to be admitted again, but the family refused.

## 3 Discussion

CHF is an autosomal recessive (AR) disorder characterized by variable degrees of periportal fibrosis, with proliferation of irregularly shaped bile ducts. This disease is quite rare, clinically marked by portal hypertension and normal liver function. Most patients initially seek treatment for gastrointestinal bleeding or unexplained hepatosplenomegaly. CHF was first described by Dobbs in 1960 and later elaborated upon by Kerr et al.^[3,5]^ A majority of the affected patients are <10 years old.^[6]^ We reported a patient with CHF and retinal macular degeneration. No similar accounts have been published to date.

CHF is a feature of Caroli syndrome. In Caroli disease, the dilated remnants of ductal plates are limited to large interlobular bile ducts, whereas aberrant small ducts are an added feature of Caroli syndrome, contributing to CHF. Cholangitis is also to be expected in patients with Caroli disease.^[7]^

CHF largely presents in childhood, manifested as portal hypertension and/or cholangitis, and is often associated with renal malformations, such as AR-PKD, renal tubular ectasia, or other forms of cystic kidney disease.^[8]^ However, recent identification of the PKHD1 gene, implicated in AR-PKD, has confirmed that some cases of CHF are indeed due to PKHD1 mutations, found in 32.1% of adults with CHF or Caroli disease.^[9]^ As we all know, CHF is a hereditary disease. We hypothesized that Some gene mutation will eventually affect the retina as it does to the lesions of liver or kidney.

Although most patients with retinal macular degeneration are adults >45 years old, its onset in this patient (at 6 years old) was unusual. Macular degeneration is due to irreversible retinal accumulation of toxic retinoid species, resulting in progressive dysfunction and loss of vision. It typically is seen in genetically normal patients as they age (age-related macular degeneration) or in adolescents with inherited genetic mutations (juvenile macular degeneration or Stargardt disease).^[10]^ In adults, there is progressive change in macular structure. Juvenile macular degeneration (also known as congenital macular degeneration) is otherwise rare in clinical practice. Like CHF, juvenile macular degeneration is an AR inherited disease in which lipids are deposited in pigmented retinal epithelial cells. At present, there is no effective treatment. A few reports describe both diseases developing in tandem.

There were several challenges posed by this patient, who was first admitted to a local hospital for gingival bleeding and diagnosed with ITP. In general, splenomegaly is not a feature of ITP, and the bone marrow biopsy that we subsequently performed ruled out hematologic disease. In conjunction with other findings, we could have suspected hypersplenism as the root cause. This patient is a young male, liver and spleen enlargement has been found at an early age, liver function is normal, portal hypertension is the main clinical manifestation. Hepatitis B, hepatitis C and other etiological examination are negative. The reason for splenomegaly we believed is due to liver fibrosis.

This patient was also diagnosed at a very young age with macular degeneration. In the course of diagnosis, we repeatedly queried relatives on family marriages and genetic histories to no avail, entertaining the possibility of 2 separate congenital disorders. In scouring the relevant literature, we learned that the cause of CHF is still unknown, although sporadic familial clustering suggests that some cases may be inherited, especially if PKD and genetic mutations coexist.^[11]^

CHF is not only linked to the ciliopathies (disorders of primary cilia) and hepatorenal fibrocystic diseases (AR-PKD in particular),^[6]^ but it is also observed in a host of syndromes (Meckel-Gruber, Ivemark, Jeune, Joubert, Bardet-Biedl, and Arima).^[3,12]^ Genetic mutations have been identified in more common and better investigated conditions (e.g., Joubert and Bardet-Biedl syndromes).^[7]^

Joubert syndrome (JS) is an AR disorder first reported in 1969.^[13]^ JS and related disorders (JSRD) are clinically heterogeneous diseases in which a multiplicity of neurologic signs and organs are involved. The retina, kidneys, liver, and skeleton are primarily affected. This marked pleiotropism is likely explained by the various genetic defects encountered. The retina is one of the most frequently affected organs in JSRD, usually in the form of retinal dystrophy due to progressive degeneration of photoreceptor cells.

Bardet-Biedl syndrome (BBS) is a clinically and genetically heterogeneous AR ciliopathy first detailed by the ophthalmologists Laurence and Moon (1866). Its chief clinical features are rod-cone dystrophy, polydactyly or dystrophic digits (brachydactyly or syndactyly), obesity, reduced intelligence, renal dysfunction, and male hypogonadism. Aside from polydactyly (a congenital finding), these features emerge in the first decade of life. Although hepatic fibrosis is a common secondary feature,^[14]^ rod-cone dystrophy (90–100% prevalence) is clearly the most important element for a diagnosis of BBS. Early-onset, severe, and progressive retinal dystrophy due to dysfunctional rods and cones is consistently reported.^[15]^ An AR mode of inheritance is usually observed, although oligogenic inheritance has been cited on occasion.^[16]^

Asphyxiating thoracic dystrophy, also known as Jeune syndrome, is an AR chondrodysplasia characterized by a narrow thorax, trident acetabular roof, polydactyly, and short limbs. Such patients are also potentially subject to renal, hepatic, pancreatic, and retinal manifestations during the course of the disease.^[17]^ Jeune syndrome belongs to a group of syndromic skeletal ciliopathies due to mutated genes encoding proteins required for formation or function of motile cilia. Cilia are components of nearly all vertebrate cells, so their dysfunction inflicts a myriad of ailments, typically retinal degeneration, renal disease, and cerebral anomalies. CHF, diabetes, obesity, and skeletal dysplasias may also develop.^[18]^

To date, ciliopathic defects have been traced to mutations in >40 genes. The patient described herein displayed many clinical findings suspicious of genetic origins, namely retinal macular degeneration, a sixth toe on the left foot, and hypogonadism. So, we thought that the above symptoms of this patient may be a part of a syndrome. Since the patient and his family refused to take a genetic test, the conjecture could not be confirmed in the patient. We suggested further genetic testing, but the family refused.

CHF is a hereditary disease of low incidence, the studies of which have focused mainly on liver involvement. It is easy to ignore that other organs, such as the retina, are part of a broader disease complex. We believe it reasonable to genetically screen patients presenting with unexplained portal hypertension and suspected CHF. In young patients, causes of noncirrhotic portal hypertension are elusive. As the presented case illustrates, liver fibrosis in some patients may have genetic underpinnings.

CHF is a disorder which is characterized by well-preserved liver function with a rather good prognosis, when complications, such as portal hypertension and variceal bleeding are controlled. However, the natural history of CHF is variable, making it difficult to prognose the outcome. As in our patient, progressive fibrosis may result in portal hypertension with variceal bleeding. Mutation analysis may provide useful information for clinicians to develop clinically relevant prognostic markers.

Pathologic examination is the diagnostic gold standard for CHF. Liver transplantation is the only treatment option. No therapies will repair primary ductal plate malformations, reverse the fibrosis, or resolve aberrant biliary trees. In children with coexisting end-stage renal failure, as a result of polycystic kidney disease, combined liver and kidney transplantation should be considered.^[4]^

## Author contributions

**Conceptualization:** Dezhao Li, Xu Li.

**Funding acquisition:** Xu Li.

**Resources:** Xu Li.

**Visualization:** Xu Li.

**Writing – original draft:** Dezhao Li, Xu Li, Shijuan Sun.

**Writing – review & editing:** Xu Li, Junjie Qin.
